# Signalling mechanisms in PAF-induced intestinal failure

**DOI:** 10.1038/s41598-017-13850-x

**Published:** 2017-10-17

**Authors:** Ingmar Lautenschläger, Yuk Lung Wong, Jürgen Sarau, Torsten Goldmann, Karina Zitta, Martin Albrecht, Inéz Frerichs, Norbert Weiler, Stefan Uhlig

**Affiliations:** 1 Department of Anaesthesiology and Intensive Care Medicine, University Medical Centre Schleswig-Holstein, Campus Kiel, Kiel Germany; 2 Division of Mucosal Immunology and Diagnostic, Research Centre Borstel, Leibniz-Centre for Medicine and Biosciences, Borstel, Germany; 3 Division of Clinical and Experimental Pathology, Research Centre Borstel, Leibniz-Centre for Medicine and Biosciences, Borstel, Germany; 4 Institute of Pharmacology and Toxicology, Medical Faculty, RWTH Aachen University, Aachen, Germany

## Abstract

Capillary leakage syndrome, vasomotor disturbances and gut atony are common clinical problems in intensive care medicine. Various inflammatory mediators and signalling pathways are involved in these pathophysiological alterations among them platelet-activating factor (PAF). The related signalling mechanisms of the PAF-induced dysfunctions are only poorly understood. Here we used the model of the isolated perfused rat small intestine to analyse the role of calcium (using calcium deprivation, IP-receptor blockade (2-APB)), cAMP (PDE-inhibition plus AC activator), myosin light chain kinase (inhibitor ML-7) and Rho-kinase (inhibitor Y27632) in the following PAF-induced malfunctions: vasoconstriction, capillary and mucosal leakage, oedema formation, malabsorption and atony. Among these, the PAF-induced vasoconstriction and hyperpermeability appear to be governed by similar mechanisms that involve IP3 receptors, extracellular calcium and the Rho-kinase. Our findings further suggest that cAMP-elevating treatments – while effective against hypertension and oedema – bear the risk of dysmotility and reduced nutrient uptake. Agents such as 2-APB or Y27632, on the other hand, showed no negative side effects and improved most of the PAF-induced malfunctions suggesting that their therapeutic usefulness should be explored.

## Introduction

Vasoconstriction and vasoplegia, endothelial and epithelial hyperpermeability^1^, tissue oedema formation with organ and body weight gain and in the end paralytic ileus with intestinal failure are typical characteristics of sepsis, especially if the gastrointestinal tract is involved^2^. Platelet-activating factor (PAF) is among those mediators that cause similar symptoms^3,4^ and that have been implicated in inflammatory bowel diseases^5–7^.

PAF binds to a G-protein coupled receptor^8^ that stimulates various second messenger systems^9–11^. However, the different effects of PAF, namely vasoconstriction or vasoplegia (dependent on the endothelial segment), vascular and mucosal hyperpermeability, oedema formation, and gut atony^12,13^ are differentially regulated, depending on the organ and the cell type^10^. To study the effects of PAF in the intestine, we have developed a sophisticated model of the isolated perfused small intestine that allows to study many of the PAF-induced disturbances in the intestine: vasoconstriction, capillary and mucosal leakage, oedema formation, malabsorption and atony. Using this model we have shown that these pathophysiological alterations occur independent of leukocytes. They are largely independent of arachidonic-acid metabolites and insensitive to steroids. Interestingly, PAF-induced intestinal failure can be largely prevented by the vascular administration of quinidine^12^, similar to the effects of quinolines in the lungs^14,15^.

It is known that quinolines alter intracellular cation concentrations and reduce calcium oscillations or calcium sensitivity, probably by interaction with cationic channels^16,17^ or IP3 receptors^18,19^. Furthermore, the sensitivity to quinoline derivates is modulated by the intracellular cyclic nucleotide content^20,21^, while quinolines themselves may interact with phosphodiesterases^22^. Moreover, quinolines may modulate the activity of crucial regulatory kinases, among them myosin light chain kinase (MLCK) and protein kinase C (PKC)^23,24^. Notably, all these factors, i.e. calcium, cAMP, myosin light chain kinase (MLCK), myosin light chain phosphatase (MLCP), Rho-kinase and PKC, are also well known as regulators of barrier integrity, vasomotor responses, and gut atony^25–31^.

Thus, in this mechanistic study we used the isolated perfused intestine model^13^ to examine whether these pathways are involved in the PAF-induced intestinal failure.

## Results

The present data have been obtained at the same time with previously published work^12^. Therefore, the data for the PAF and the control groups are the same as before where we have confirmed our original observation^13^ that PAF leads to profound intestinal disorders characterized by strong vasoconstriction, dramatic loss of vascular volume, increase in vascular and epithelial permeability, increased oedema formation (i.e. organ weight), malabsorption and paralysis^12^.

### Calcium and cAMP

**Figure 1:**
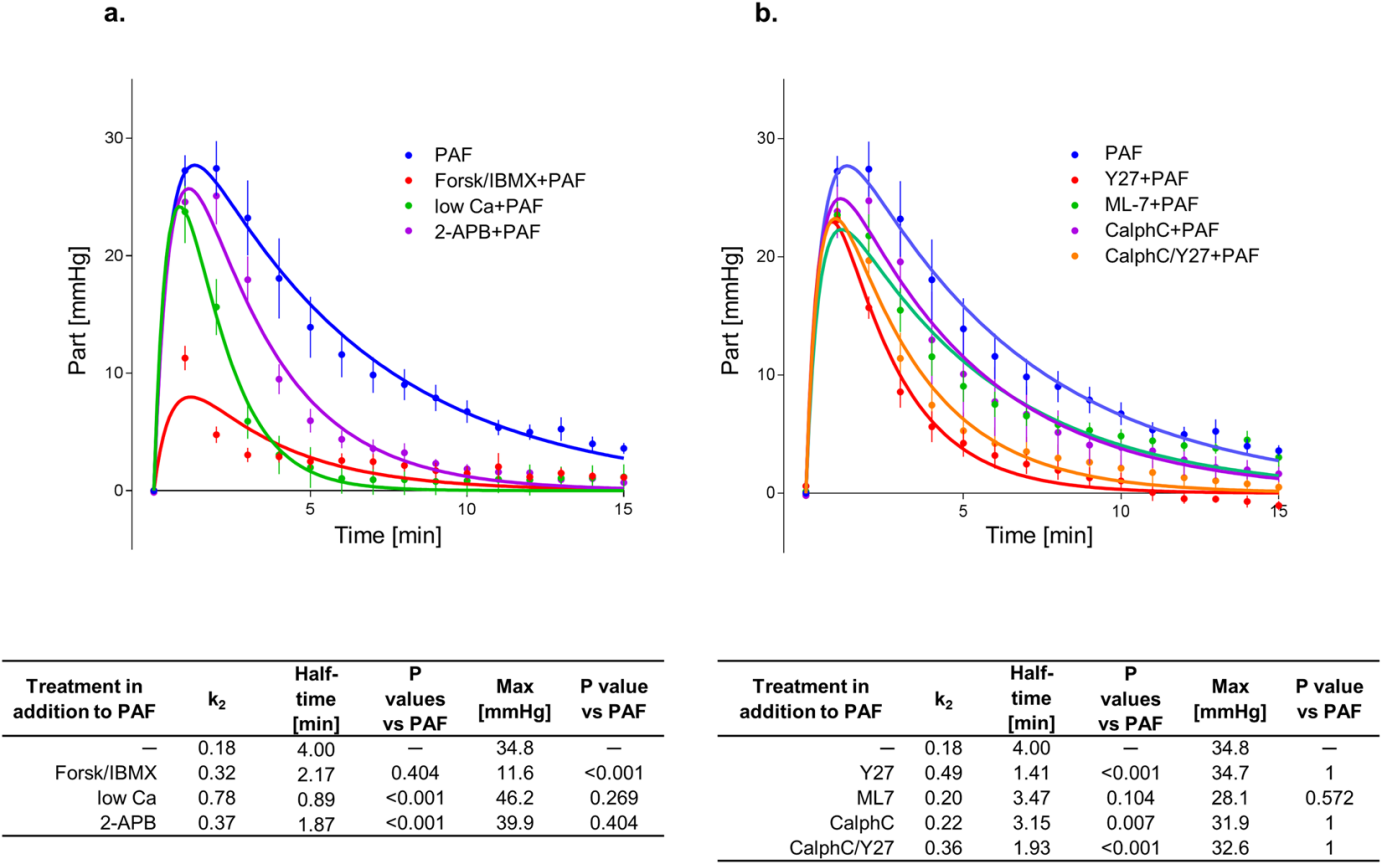
PAF-induced vasoconstriction. Intestines were stimulated with a bolus of 0.5 nmol PAF (n = 5). (**a**) Intracellular second messengers: AC stimulator forskolin plus the PDE inhibitor IBMX (Forsk/IBMX + PAF, n = 4), lowered calcium concentration (low Ca + PAF, n = 3), IP_3_ antagonist 2-APB (2-APB + PAF, n = 5), (**b**) MLC interacting kinases: Rho-kinase inhibitor Y27632 (Y27 + PAF, n = 3), myosin light chain kinase inhibitor ML-7 (ML-7 + PAF, n = 5), protein kinase C inhibitor calphostin C (CalphC + PAF, n = 3), or Rho-kinase and protein kinase C inhibitor (CalphC/Y27 + PAF, n = 3). Vascular pressure tracings of controls (n = 5), or controls with lowered calcium concentration (low Ca control, n = 4) did remain at baseline and are not shown. Using non-linear mixed modelling (Proc NLMIXED) the pressure data were analysed by a bi-exponential model with the following terms: k_1_ reflecting the rapid initial increase, k_2_ reflecting the slower decay in arterial pressure (Part) and the maximum response (M) reflecting the maximum response. None of the drugs altered k_1_ which equated to a half-time of about 30 seconds under all conditions. Forskolin plus IBMX was the only treatment that reduced the maximum pressure gain. Five treatments (low Ca, 2-APB, Y27, CalphC, CalphC/Y27) reduced the length of the second phase of the PAF-induced increase in mesenteric artery pressure. P values were adjusted for multiple comparisons by the Bonferroni-Holm procedure.

**Figure 2:**
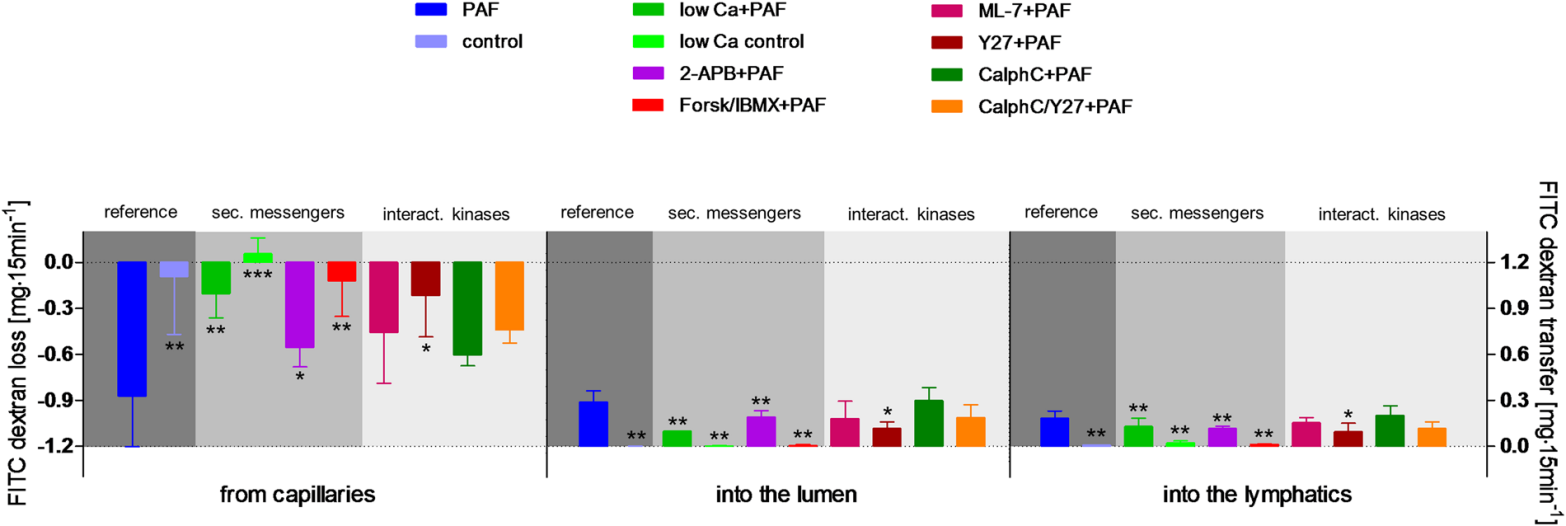
PAF-induced hyperpermeability. Effects of intracellular second messengers and MLC interacting kinases. FITC dextran loss and transfer in controls (control, n = 5), in controls with lowered calcium concentration (low Ca control, n = 4), in response to platelet-activating factor alone (PAF, n = 5) or in parallel with pretreatment by lowered calcium concentration (low Ca + PAF, n = 3), IP_3_ antagonist 2-APB (2-APB + PAF, n = 5), AC stimulator forskolin and PDE inhibitor IBMX (Forsk/IBMX + PAF, n = 4), myosin light chain kinase inhibitor ML-7 (ML-7 + PAF, n = 5), Rho-kinase inhibitor Y27632 (Y27 + PAF, n = 3), protein kinase C inhibitor calphostin C (CalphC + PAF, n = 3), or Rho-kinase and protein kinase C inhibitor (CalphC/Y27 + PAF, n = 3). Bars at the left side “from capillaries”: loss of FITC dextran from vascular circulation, bars in the middle “into the lumen”: FITC dextran transfer into the lumen, bars at the right side “into the lymphatics”: FITC dextran transfer into the lymphatics. Dark grey shaded areas: Reference PAF and control. Middle grey shaded areas: measurements in isolated intestines pretreated targeting intracellular second messengers calcium and cAMP. Light grey shaded areas: measurements in isolated intestines pretreated targeting MLC interacting kinases. Data were analysed by two-way ANOVA (factors being treatment and compartment) and P values were adjusted for multiple comparisons by the step-down Dunnett test: *p < 0.05 versus PAF; **p < 0.01 versus PAF; ***p < 0.0001 versus PAF. Abbreviations: sec. messengers = second messengers calcium and cAMP; interact. kinases = MLC interacting kinases.

**Figure 3:**
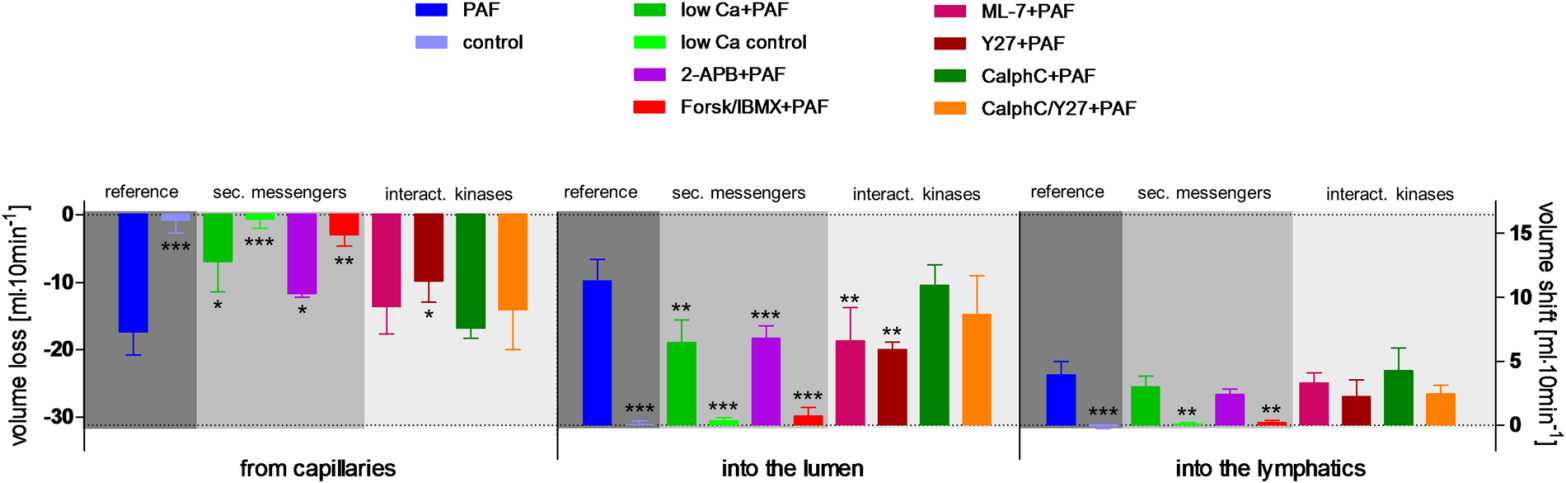
PAF-induced fluid shifts. Effects of intracellular second messengers and MLC interacting kinases. Fluid distribution in controls (control, n = 5), in controls with lowered calcium concentration (low Ca control, n = 4), in response to platelet-activating factor alone (PAF, n = 5) or in parallel with pretreatment by lowered calcium concentration (low Ca + PAF, n = 3), IP_3_ antagonist 2-APB (2-APB + PAF, n = 5), AC stimulator forskolin and PDE inhibitor IBMX (Forsk/IBMX + PAF, n = 4), myosin light chain kinase inhibitor ML-7 (ML-7 + PAF, n = 5), Rho-kinase inhibitor Y27632 (Y27 + PAF, n = 3), protein kinase C inhibitor calphostin C (CalphC + PAF, n = 3), or Rho-kinase and protein kinase C inhibitor (CalphC/Y27 + PAF, n = 3). Bars at the left side “from capillaries”: loss of fluid from vascular circulation, in the middle “into the lumen”: volume shift into the lumen, bars at the right side “into the lymphatics”: volume shift into the lymphatics. Dark grey shaded area: Reference PAF and control. Middle grey shaded area: measurements in isolated intestines pretreated targeting intracellular second messengers calcium and cAMP. Light grey shaded area: measurements in isolated intestines pretreated targeting MLC interacting kinases. Data were analysed by two-way ANOVA (factors being treatment and compartment) and P values were adjusted for multiple comparisons by the step-down Dunnett test: *p < 0.05 versus PAF; **p < 0.01 versus PAF; ***p < 0.0001 versus PAF. Abbreviations: sec. messengers = second messengers calcium and cAMP; interact. kinases = MLC interacting kinases.

**Figure 4:**
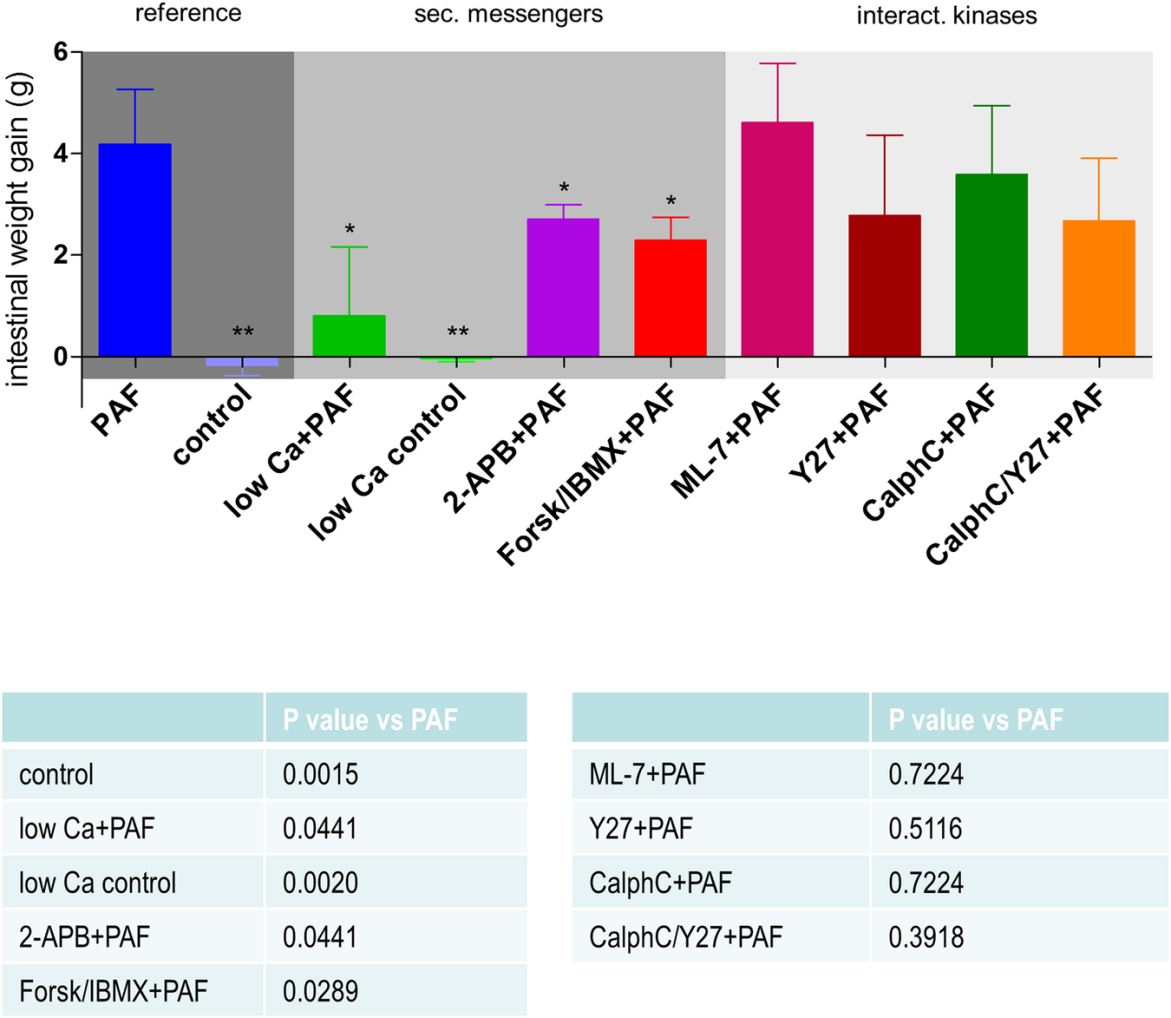
PAF-induced intestinal weight gain. Weight gain (210 seconds after stimulation) in controls (control, n = 5), in controls with lowered calcium concentration (low Ca control, n = 4), in response to platelet-activating factor alone (PAF, n = 5) or in parallel with pretreatment by lowered calcium concentration (low Ca + PAF, n = 3), IP_3_ antagonist 2-APB (2-APB + PAF, n = 5), AC stimulator forskolin and PDE inhibitor IBMX (Forsk/IBMX + PAF, n = 4), myosin light chain kinase inhibitor ML-7 (ML-7 + PAF, n = 5), Rho-kinase inhibitor Y27632 (Y27 + PAF, n = 3), protein kinase C inhibitor calphostin C (CalphC + PAF, n = 3), or Rho-kinase and protein kinase C inhibitor (CalphC/Y27 + PAF, n = 3). Dark grey shaded area: Reference PAF and control. Middle grey shaded area: measurements in isolated intestines pretreated targeting intracellular second messengers calcium and cAMP. Light grey shaded area: measurements in isolated intestines pretreated targeting MLC interacting kinases. Abbreviations: sec. messengers = second messengers calcium and cAMP; interact. kinases = MLC interacting kinases. P values: *p < 0.05 versus PAF; **p < 0.01 versus PAF; ***p < 0.0001 versus PAF.

**Figure 5:**
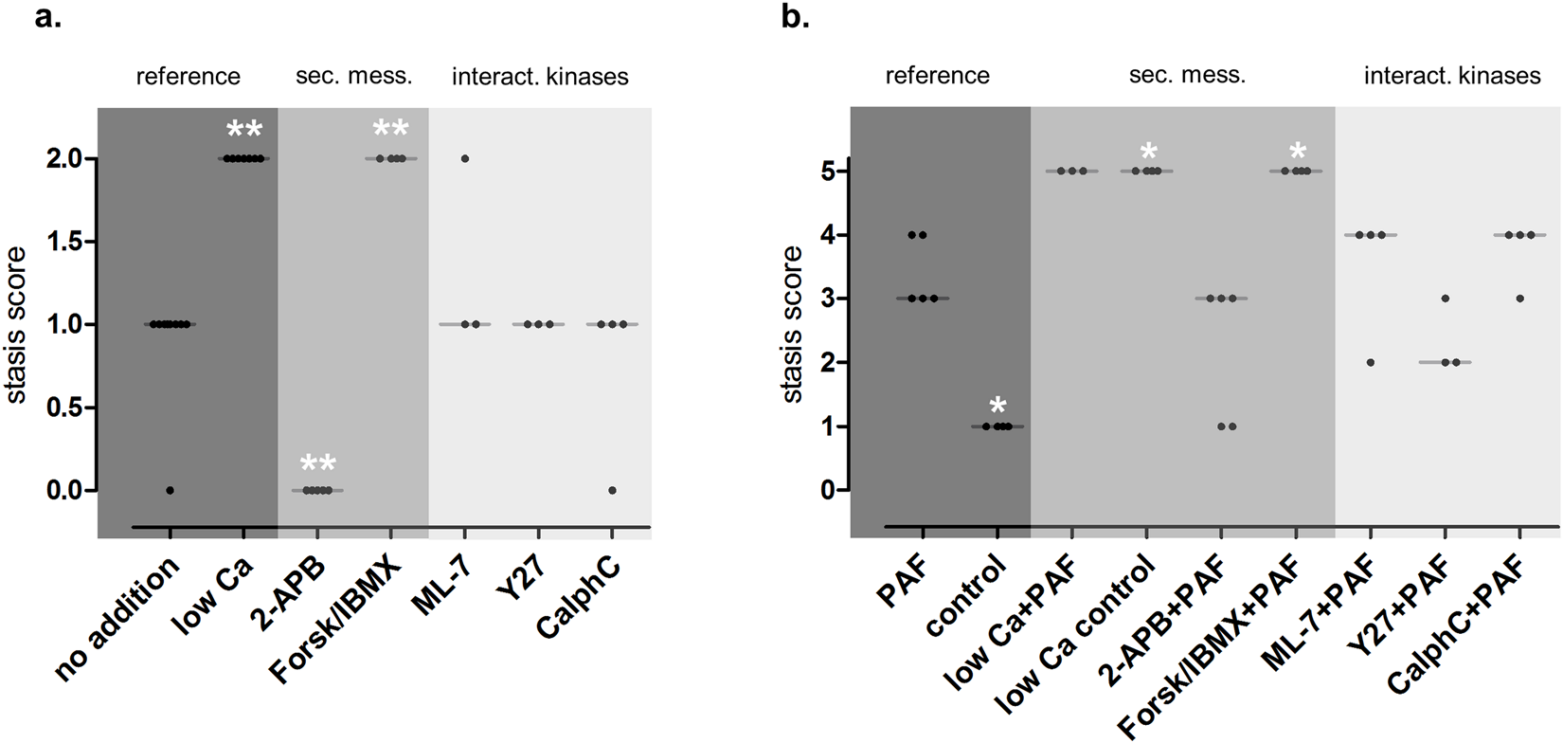
Peristalsis. (**a**) Peristalsis score without substance addition or after initiation of pretreatment, before stimulation with PAF. Scoring: 0 = prokinetic, 1 = normal (unchanged), 2 = stasis. Peristalsis score of intestines without substance addition (control, n = 4 and PAF group, n = 5 before PAF are plotted together and labelled “no addition”), in controls with lowered calcium concentration (low Ca control, n = 4 and low Ca + PAF, n = 3 before PAF are plotted together and labelled “low Ca”), and after the initiation of pretreatment with 2-APB (2-APB, n = 5), with forskolin and IBMX (Forsk/IBMX, n = 4), with ML-7 (ML-7, n = 5), with Y27632 (Y27, n = 3) and with calphostin C (CalphC, n = 3) are shown. (**b**) Peristalsis score after stimulation with PAF. Scoring: 0 = prokinetic, 1 = normal, 2 = stasis with duration < 60 seconds, 3 = stasis with duration between 60 to 120 seconds, 4 = stasis with duration between 120 and 360 seconds, 5 = permanent stasis. Peristalsis score in response to platelet-activating factor alone (PAF, n = 5) or in parallel with pretreatment by lowered calcium concentration (low Ca + PAF, n = 3), IP_3_ antagonist 2-APB (2-APB + PAF, n = 5), AC stimulator forskolin and PDE inhibitor IBMX (Forsk/IBMX + PAF, n = 4), myosin light chain kinase inhibitor ML-7 (ML-7 + PAF, n = 5), Rho-kinase inhibitor Y27632 (Y27 + PAF, n = 3), or protein kinase C inhibitor calphostin C (CalphC + PAF, n = 3) as well as in controls (control, n = 4) are shown. Analysis of the group CalphC/Y27 + PAF was not performed due to technical problems with video capturing in some experiments. Scores were analysed by two-sided Mann-Whitney-tests and the p values were corrected by the Hommel procedure (Proc Multtest): *p < 0.05 versus PAF; **p < 0.01 versus PAF. Data are shown as dots plots with median (line).

**Figure 6:**
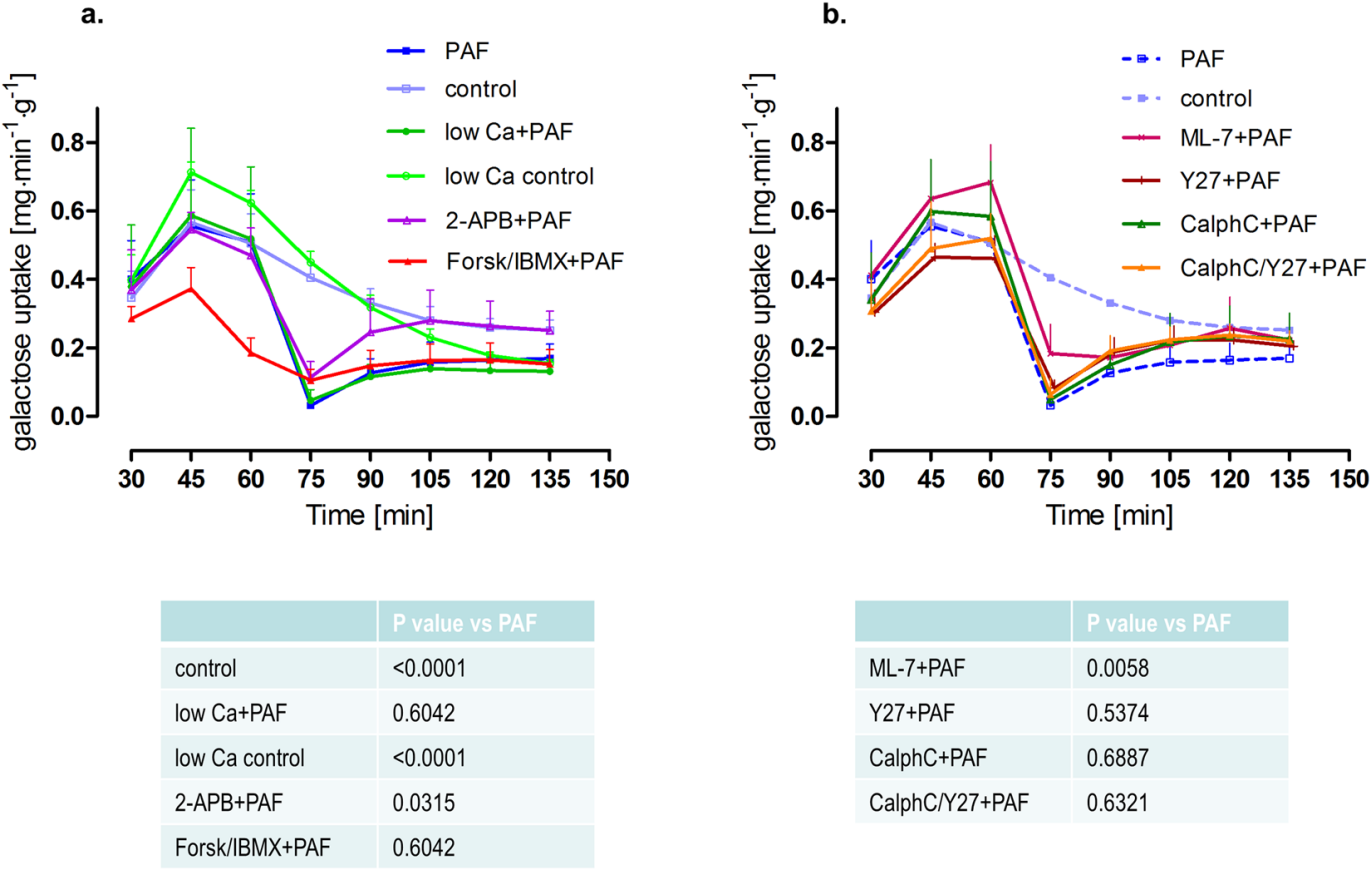
Galactose uptake. Tracings of galactose uptake in controls (control, n = 5), in controls with lowered calcium concentration (low Ca control, n = 4), in response to platelet-activating factor alone (PAF, n = 5) or in parallel with pretreatment by lowered calcium concentration (low Ca + PAF, n = 3), IP_3_ antagonist 2-APB (2-APB + PAF, n = 5), AC stimulator forskolin and PDE inhibitor IBMX (Forsk/IBMX + PAF, n = 4), myosin light chain kinase inhibitor ML-7 (ML-7 + PAF, n = 5), Rho-kinase inhibitor Y27632 (Y27 + PAF, n = 3), protein kinase C inhibitor calphostin C (CalphC + PAF, n = 3), or Rho-kinase and protein kinase C inhibitor (CalphC/Y27 + PAF, n = 3). (**a**) Groups with pretreatment targeting second messengers calcium and cAMP. (**b**) Groups with pretreatment targeting MLC interacting kinases. Data were analysed at t = 75 min by one-way ANOVA and the P-values adjusted by the step-down Dunnett procedure.

Reducing the extracellular calcium concentration to one-tenth of the physiological level (0.25 nM) reduced the half-time of the second phase of the PAF-induced vasoconstriction from 4 min to 53 seconds, but had no effect on the maximum mesenteric artery pressure (Fig. 1a). Low calcium levels further attenuated the permeability of the vasculature to FITC dextran (Fig. 2), the volume shift from the vessels into the lymphatics and the lumen (Fig. 3), and the intestinal weight gain (Fig. 4). On the other hand, the calcium restriction impaired physiological peristalsis (Fig. 5a) and failed to improve PAF-induced motility disorders (Fig. 5b). The PAF-induced loss of galactose uptake was not improved by reduction of extracellular calcium (Fig. 6a).

Inhibition of calcium release from intracellular stores by the IP3-receptor antagonist (2-APB) almost halved the second phase of the PAF-induced vasoconstriction (half-time = 1.9 min vs 4 min) (Fig. 1). It also reduced the permeability changes and the fluid shifts, albeit not as potently as the extracellular calcium reduction (Figs 2–4). Notably, however, 2-APB improved peristalsis before and by trend after PAF administration (Fig. 5a,b). Furthermore, the recovery from PAF-induced loss of galactose uptake was slightly faster (Fig. 6).

Stimulation of cAMP production by the combination forskolin/IBMX reduced the maximum increase in mesenteric artery pressure from 34.8 mmHg to 11.6 mmHg (Fig. 1a), but had no effect on the recovery phase. This treatment also potently reduced the extravasation of FITC dextran to the lumen and the lymphatics (Fig. 2), the fluid shift from the circulation and the intestinal weight gain (Fig. 4). Similar to the effects of calcium restriction, cAMP elevation also reduced peristalsis (Fig. 5) and galactose uptake even before PAF was given (Fig. 6).

### Kinases

**Figure 7:**
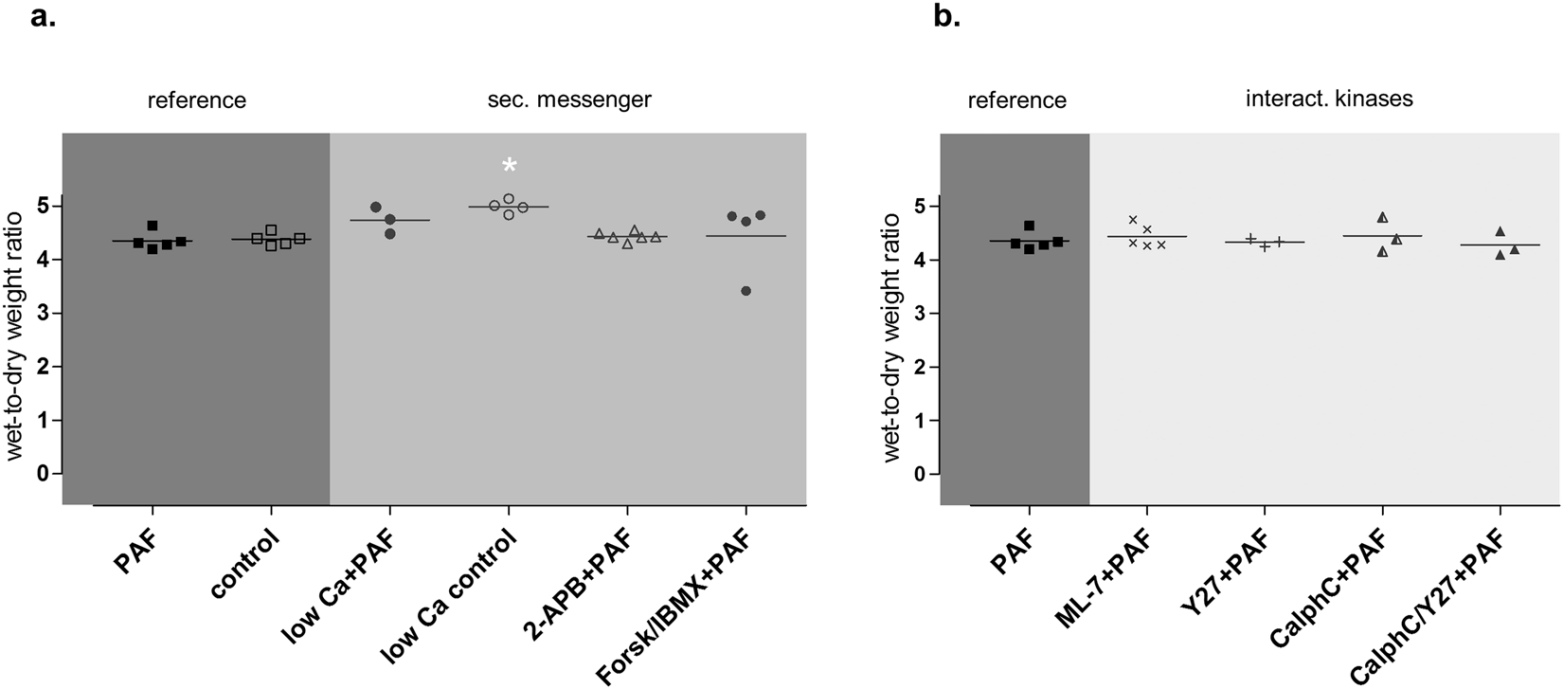
Wet-to-dry weight ratio. Wet-to-dry weight ratio was calculated from intestinal weight at the end of the experiments. (**a**) Groups with pretreatment targeting second messengers calcium and cAMP. (**b**) Groups with pretreatment targeting MLC interacting kinases. *p < 0.05 versus PAF. Data are shown as dots plots with median (line).

**Figure 8:**
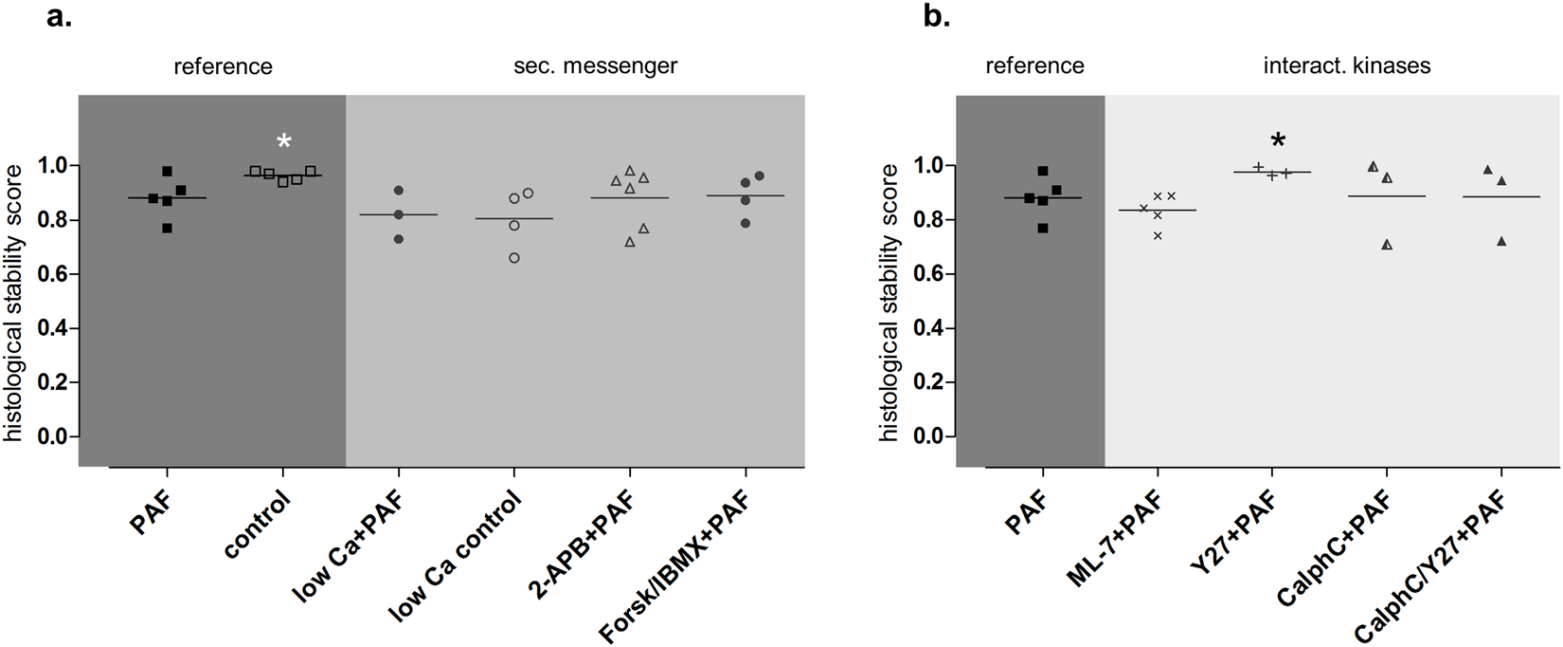
Histological stability score. (**a)** Groups with pretreatment targeting second messengers calcium and cAMP. (**b**) Groups with pretreatment targeting MLC interacting kinases. *p < 0.05 versus PAF. Data are shown as dots plots with median (line).

Inhibition of the myosin light chain kinase (MLCK) with the MLCK-inhibitor ML-7 failed to prevent the PAF-induced vasoconstriction (Fig. 1b), the permeability increase (Fig. 2), the volume shift (Fig. 3), the weight gain (Fig. 4), the dysmotility (Fig. 5b), and the impaired galactose uptake (Fig. 6b). ML-7 did not alter the wet-to-dry weight ratio (Fig. 7b) or histological stability score (Fig. 8b).

Inhibition of the Rho-kinase by Y27632 did not alter the maximum vasoconstriction, but decreased the half-time of the second phase from 4 min to 1.4 min (Fig. 1b). It reduced the loss of the extravasation of FITC dextran to the lumen and the lymphatics (Fig. 2), the fluid shift from the circulation (Fig. 3), while the intestinal weight gain remained unchanged (Fig. 4). Interestingly, the PAF-induced reduction in peristalsis was diminished and the recovery to normal motility was faster (Fig. 5b), while Y27632 did not influence baseline motility (Fig. 5a). The PAF-induced loss of galactose uptake was not prevented (Fig. 6). Of note, the histological stability score was the highest in this group (Fig. 8b), the intestinal villi appeared as tall as the usual villus architecture (Supplementary Fig. S1) and the wet-to-dry weight ratio was normal at the end of the experiments (Fig. 7b).

Inhibition of protein kinase C by the fungus metabolite calphostin C slightly reduced the second phase of the PAF-induced vasoconstriction (Fig. 1b). There were no significant changes in PAF-induced changes in permeability (Fig. 2), fluid shifts (Fig. 3), weight gain (Fig. 4), dysmotility (Fig. 5b) and loss of galactose uptake (Fig. 6). The wet-to-dry weight ratio (Fig. 7b) and the histological stability score were normal (Fig. 8b) and the baseline peristalsis score before PAF administration was comparable to controls (Fig. 5a). The dual inhibition of protein kinase C and Rho-kinase appeared to be somewhat less effective in protection from PAF-induced intestinal disorders than inhibition of Rho-kinase alone (Figs 1–8).

In order to improve our understanding of how the burden of increased vascular permeability is shared between the lymph and the lumen under the various conditions, we looked at the statistical interaction between the treatments and the compartments (lymph, lumen) using a 2-way ANOVA. The absence of a significant interaction term (treatment x compartment) for FITC dextran suggests that the distribution of FITC dextran between lymph and lumen depends on the same factor – probably the passage through the endothelial barrier. For the volume shift there was a positive statistical interaction effect (p < 0.05) between treatments and compartments, indicating that there was relatively less fluid shift into the lumen in case of small oedema and a relatively larger fluid shift into the lumen in case of stronger oedema. This interaction may indicate that the increased hydrostatic pressure – and possibly also the altered motility – following the PAF administration affects the luminal efflux more than the lymph flow.

All treatments were well tolerated as indicated by the wet-to-dry weight ratios (Fig. 7) and the histological stability scores (Fig. 8) that were obtained at the end of the experiments. The only effects that were noted were a small increase in the wet-to-dry weight ratio and a slight shortening of the villi length in the low calcium control group (Supplementary Fig. S1).

## Discussion

In this study we have examined several of the major signalling pathways that are commonly associated with smooth muscle contraction and oedema formation (Table 1). Our findings suggest that calcium, partly derived from IP3-sensitive stores and to some extent the Rho-kinase pathway mediate the second phase of the PAF-induced vasoconstriction and the increased vascular permeability. The mechanisms responsible for the motility disorder and the reduced galactose uptake are much less clear, and appear to occur largely independent from any of the mechanisms studied here.

**Table 1:** Effects of the various treatments on the PAF-induced dysfunctions.

principle	treatment	vasoconstriction	hyper-permeability	hypo-motility	galactose uptake
extra-cellular calcium	1/10 calcium	↓ (2^nd^ phase)	↓	↑ (basal)	─
IP3	2-APB	↓ (2^nd^ phase)	↓	↓	↑
cAMP	forskolin/IBMX	↓ (maximum)	↓	↑ (basal)	↓ (basal)
Rho-kinase	Y27632	↓ (2^nd^ phase)	↓	↓	─
MLCK	ML-7	─	─	─	─
PKC	Calphostin C	↓ (2^nd^ phase)	─	─	─

### The isolated perfused small intestine as an appropriate model for inflammatory intestinal failure

Here we have used a comprehensive model of the isolated perfused intestine that was developed to give simultaneous access to several important aspects of the complex gut physiology: fluid balance, barrier integrity, digestion and nutrient absorption. This model offers the opportunity to detect effects and side effects at the same time, as is illustrated here for cAMP elevation that, on the one hand, decreased oedema formation and improved perfusion but, on the other hand, also promoted motility disorder and malabsorption. Such side effects would have been missed in reductionist models like cell culture and overlooked *in vivo* where all of these parameters are usually not accessible in the same experiment. It should be kept in mind that this paper examines the single-bolus effects within an *ex vivo* organ model, whereas *in vivo* mediators like PAF exert their action for a longer duration. The primary scope of this work was mechanistic: the bolus injection and the prophylactic treatment allowed us to examine the mechanisms immediately after PAF-receptor activation, among them the two phases of the vasoconstriction. The therapeutic usefulness of the successful interventions needs to be explored in further studies, including studies in intact animals.

### PAF-induced vasoconstriction

PAF is an inflammatory mediator that is believed to contribute to the small intestinal hypoperfusion and bowel necrosis during sepsis and other inflammatory disorders^32–34^. The responsible molecular mechanisms have been largely unexplored; so far it has been shown that this response occurs independent of eicosanoids^12^ and may be mitigated by the neuronal NO–synthase (nNOS)^35^.

In general, vasoconstriction depends on an increase in cytosolic calcium that in many cases – at least after stimulation of G-protein coupled receptors – occurs in two phases, an initial increase from internal stores and a second wave derived from extracellular calcium^36^. Such a biphasic response has been described for PAF in human neutrophils^37^, murine macrophages^38^ and cow tracheal epithelium^39^. Because the mesenteric artery pressure tracings also show such a biphasic response, it seems likely that these two phases reflect the cytosolic calcium concentrations. To analyse this biphasic response, we have used non-linear mixed modelling, an approach that permits the use of non-linear equations to model a given response in conjunction with the possibility to separate the fixed treatment effects from the random effects of the individual preparations. The biphasic exponential equation that we utilized – also known as the Bateman equation – explained about 84% of the data (R^2^), and can thus be considered a useful mathematical description of the vasoconstriction kinetics. This analysis revealed that the first phase has a half-time of about 30 seconds and the second a half-time of 4 min, which is comparable to the effects of PAF in tracheal epithelial cells^39^. To our knowledge this is first time that non-linear mixed modelling has been applied to vascular smooth muscle responses and we believe that the ability to discriminate the maximum response and the two phases encourages further use of this method.

In order to explore the role of extracellular calcium, we perfused the intestines with 1/10 of the normal calcium concentrations. In line with our hypothesis, this treatment had no effect on the first, but largely prevented the second phase, indicating that these two phases of vasoconstriction do indeed depend on internal and external calcium, respectively. Our findings with 2-APB and forskolin/IBMX suggest that the first phase is blocked by cAMP and is not dependent on IP3-sensitive stores; possible mechanisms include activation of the epac/Rap1 pathway^40^ or PKA-dependent activation of the sarcoplasmic/endoplasmic reticulum calcium ATPase (SERCA)^41^. The second phase appears to depend largely on both IP3 (blocked by 2-APB) and the Rho-kinase pathway (blocked by Y27632). These findings are in line with the concept that Rho-kinase is activated by membrane potential depolarization and calcium release from internal stores – in this case mobilized by IP3^42^. Because Y27632 may also block PKC^43^ and because PKC has itself been involved in vascular smooth muscle contraction^44^, we studied the effects of calphostin C. This drug showed only a small effect on the second phase and was not additive to Y27632. Overall, these findings suggest that the majority of the effects of Y27632 were due to its effects on Rho-kinase. A critical role of Rho-kinase with little or no role for the MLCK is in line with the mechanisms of PAF-induced vasoconstriction in the lungs^45^, although this similarity may be superficial because, in contrast to the intestine^12^, the PAF-induced pressor responses in the lungs occur also in the arteries and depend largely on eicosanoids^46^.

Taken together with our previous findings, we propose that PAF stimulates PAF-receptors in the mesenteric veins^12^ that elicit a rapid contraction mediated by an unknown cAMP-sensitive mechanism. The second phase appears to depend partly on IP3-sensitive internal calcium stores that trigger the influx of extracellular calcium – probably through voltage and/or store operated calcium channels – that in turn leads to Rho-kinase activation and sustained vasoconstriction.

### PAF-induced oedema formation

Intestinal oedema formation is a critical complication in sepsis^3,4^ when inflammatory mediators such as PAF increase vascular permeability to large molecules and water. In the present study permeability was assessed by high-molecular FITC dextran that was preferred over FITC albumin in order to exclude transcytosis^47^. In principle, PAF might also increase the epithelial barrier permeability^48^. Therefore, it was important that the 2-way ANOVA indicated that the distribution of FITC dextran between lymph and lumen was not affected by any of the treatments, suggesting either that the endothelial and epithelial macromolecular permeability are governed by the same factors or that alterations in the mucosal permeability to macromolecules do not play an important role in this model. The overall permeability to water was examined by constantly weighing the intestine and in further detail by the flux of water from the perfusate to the lymph and the lumen. The fluid shift analysis accounted for about 80% of the total organ weight changes; the unaccounted 20% may be explained by the varying volume of vessels and lumen due to varying vascular tone. Our findings indicate that the FITC dextran measurements reflect the endothelial permeability, whereas the volume shifts represent the sum of vascular leakage, hydrostatic forces and gut motility.

The gap formation between endothelial cells – the basis for increased vascular permeability – is also thought to depend on calcium. In line with this, PAF is known to increase calcium in mesenteric endothelial cells^49^. Previous studies on the mechanisms of PAF-induced oedema formation in the intestine had shown that this process depends on nitric oxide^49,50^ and on cadherins^51,52^, but is independent of MLCK^53,54^ and Rho-kinase^55^. Our FITC dextran data now extend these findings by directly demonstrating the importance of extracellular calcium and by showing a small, but significant effect of IP3 receptors (2-APB data). The protection seen with 10 µM Y27632 in the present study is in contrast to those in isolated mesenteric veins, where 30 µM Y27632 had no effect on the PAF-induced permeability. There are at least two (not mutually exclusive) possibilities to explain this important discrepancy: (i) Although, in general, permeability oedema is thought to occur in postcapillary venules^56^, part of the leakage may occur at distinct sites such as in the capillaries^57^. (ii) The vascular permeability is enhanced by hydrostatic mechanisms. Such a mechanism cannot be excluded, because in the present study there was a good correlation between the ability of drugs to reduce vasoconstriction and FITC dextran measurements.

The most effective protection against the PAF-induced hyperpermeability was provided by forskolin/IBMX. It is well known that cAMP-enhancing drugs stabilize oedema formation and, at least in the case of PAF, the related mechanism occurs most likely by PKA-independent activation of the epac/Rap1-pathway^58^. cAMP enhancing agents have been shown to be effective in models of sepsis^59,60^. However, cAMP elevation had also side effects (motility disorder, malabsorption) that may limit its clinical use (see below).

### PAF-induced motility disorder

Gastrointestinal symptoms, among them motility disorder, worsen the outcome of critically ill patients^61^. Unfortunately, early signs of motility disorder are frequently overlooked and a suitable monitoring of intestinal (motor)-function is lacking^62^. This was overcome in our model by direct inspection of the intestinal motility.

There exists no comprehensive molecular concept for the regulation of peristalsis valid for the entire alimentary tract. What is known is that gastric emptying and intestinal motility are controlled quite intricately and depend on the fine adjustments of (i) several cell types^63^, (ii) receptors^62,64^ and (iii) diverse signalling pathways^63^. These are dysregulated in inflammation and in experimental sepsis/endotoxemia models PAF is involved in dysmotility^65^ and vomiting^66^. Of relevance in this context, PAF-receptor positive cells in the enteric nervous system of guinea pigs are mostly cholinergic^67^ and in isolated perfused intestines from rabbits PAF inhibits colonic motility while tissue levels of neuropeptides are increased^68^. However, a mechanistic explanation of the PAF-induced motility disorder has not yet been provided and clinically PAF has not yet been implicated in the (therapeutic) concept of gastroparesis or paralytic ileus^62,63^. It seems likely that in critically ill patients with high levels of inflammatory mediators such as TNF or PAF^10,69,70^, intestinal paralysis may in part be provoked by these mediators and may delay recovery. We have recently shown that PAF-induced intestinal paralysis is prevented neither by eicosanoid antagonism nor by corticoids, while quinidine provided protection from PAF-induced intestinal paralysis by yet poorly understood mechanisms^12^.

Here we studied the role of calcium, cAMP and MLC-interacting kinases in PAF-induced intestinal motility. Although the inhibition of phosphodiesterase and of Rho-kinase protected from PAF-induced microcirculatory dysfunction and oedema formation, none of these treatments was clearly beneficial for intestinal motility, albeit the inhibition of IP3-receptors was prokinetic at baseline and tended to improve the recovery from PAF-induced gut atony. The findings and the lack of a molecular concept for gastrointestinal dysmotility suggest that it will remain difficult to treat inflammatory motility disturbances and to improve all aspects of intestinal failure in septic patients. We hope that our findings on quinolines, IP3-receptor antagonism and future experiments on PAF-signalling in the intestine will help to develop a more comprehensive understanding of the molecular mechanisms in the paralytic and inflamed gut.

### PAF-induced maldigestion and -absorption

The nutrient absorption is impaired in critically ill patients^71,72^ leading to malnutrition that worsens the clinical outcome^73^. In critical illness a reduction of intestinal glucose transporters was found^72^ and suggested to be the most likely mechanism for glucose malabsorption^74^, while dysmotility or abnormal disaccharide concentration in the mucosa were not considered crucial for malabsorption^75^.

The reduction of intestinal galactose transport was explained by a decreased amount of e.g. the transporter SGLT-1 in enterocytes and an involvement of PKC, PKA and of mitogen-activated protein kinases^76^ in an everted sac endotoxemia model in the rabbit. Beside LPS, also PAF has been implicated in sepsis and multiple organ dysfunction and we have demonstrated that PAF reduces galactose uptake^13^ in the isolated perfused rat small intestine. The present data now show that neither elevation of cAMP (PKA and/or epac/Rap1 activation) nor the inhibition of PKC can protect from the PAF-induced loss of galactose uptake, indicating that neither PKA activation nor PKC inhibition will be of help in stabilizing inflammatory malabsorption. Interestingly and in addition to a protective, quinidine-sensitive pathway described in our previous study^12^, the present findings suggest that IP3 inhibition may improve maldigestion and -absorption in PAF-induced intestinal inflammation. Of note, the extracellular reduction of calcium was well-tolerated and although motility was decreased, digestion and absorption was sustained. This is in line with the clinical experience that dysmotility is not a prerequisite for impaired saccharide uptake (see above). All other treatments analysed here failed to improve PAF-induced maldigestion and absorption; on the other hand, none of the treatments damaged the mucosal surface as shown by our histological analysis.

## Conclusion

Mechanistically, the PAF-induced vasoconstriction and hyperpermeability appear to be governed by similar mechanisms involving IP3 receptors, extracellular calcium and the Rho-kinase (Table 1). While this study was not designed to evaluate possible therapies, our findings suggest that cAMP-elevating treatments – while effective against hypertension and oedema – bear the risk of dysmotility and reduced nutrient uptake. Agents such as 2-APB or Y27632, on the other hand, showed no negative side effects and improved most of the PAF-induced malfunctions suggesting that their therapeutic usefulness should be explored.

## Methods

### Animals

We used 10 to 12 weeks old female Wistar rats weighing 230 ± 15 g (mean ± SD) as donors. Rats were obtained from Charles River (Charles River Laboratories, Sulzfeld, Germany). The study was conducted in agreement with the ethical requirements of the Animal Care Committee of the Ministry of Energy, Agriculture, Environment and Rural Areas of Schleswig-Holstein, Germany and in direct accordance with the German Animal Protection Law. The protocols were approved by the Ministry of Energy, Agriculture, Environment and Rural Areas of Schleswig-Holstein, Germany (Protocol: V312-72241.123-3, A47 and A56). All efforts were made to minimize suffering. The animals were anesthetized by inhalation of sevoflurane and supplemented with an intramuscular injection of ketamine. Subsequent to the dissection of the intestine, a lethal dose of pentobarbital (100 mg) was used for euthanasia.

### Study design

The experimental groups are summarized in the Supplementary Table S2 and presented schematically in the Supplementary Fig. S3. PAF was given after 60 minutes of equilibration of the perfusion model as a bolus of 0.5 nmol via the mesenteric artery within 20 seconds (PAF, n = 5); in addition we performed control experiments with normal buffer (control, n = 5) and with low calcium (0.25 mM) containing buffer (low calcium, n = 4).

All pharmacological agents were administrated 20 minutes before stimulation with PAF. In the first series of experiments, we analysed the role of the second messengers calcium and cAMP: low calcium (0.25 mM) containing perfusate (low Ca + PAF, n = 3), the IP_3_ receptor antagonist 2-APB (50–150 µM, 2-APB + PAF, n = 5), the adenylate cyclase stimulator forskolin (0.5 µM) together with the PDE-inhibitor IBMX (100 µM, Forsk/IBMX + PAF, n = 4). In the second series, we analysed the role of three kinases: the myosin light chain kinase by its blocker ML-7 (35 µM, ML-7 + PAF, n = 5), the Rho-kinase by its blocker Y27632 (10 µM, Y27 + PAF, n = 3), and the protein kinase C by its inhibitor calphostin C (0.5 µM, CalphC + PAF, n = 3). In one experimental group protein kinase C and Rho-kinase were blocked simultaneously (CalphC/Y27 + PAF, n = 3).

### Isolated perfusion and analysis

The small intestines from non-fasted rats were isolated and perfused as described in detail before^12,13,77^. Fluid shifts and macromolecular transfer of FITC-labelled dextran (150 kDa) within the vascular, lymphatic and luminal compartments of the gut were assessed and the compartment pressures were recorded simultaneously. The vascular fluid losses to the lymphatic and luminal compartments were calculated from the cumulative weight measurements of the drained fluid. The transfer of the vascular tracer (FITC dextran) as a measure of endothelial and epithelial permeability and the resorption of galactose derived from luminal lactose as a measure of metabolic competence were recorded every 15 minutes by standard photometric assays. Aerobic metabolism was ensured by measurement of lactate-to-pyruvate ratio by a standard photometric assay.

### Analysis of peristalsis

Intestinal motility was captured by video filming for offline blinded analysis. Quantification of motility was done twice to evaluate the baseline effects of the respective treatment and its potential effect against the PAF-induced stasis: first, immediately before PAF application (thus after pretreatment) and second after stimulation with PAF. Stasis score values were attributed to the motility pattern for the first interval as follows: 0 = prokinetic; 1 = normal; 2 = stasis, and for the second interval as follows: 0 = prokinetic; 1 = normal; 2 = stasis with duration < 60 seconds; 3 = stasis with duration between 60 to 119 seconds; 4 = stasis with duration between 120 and 360 seconds; 5 = permanent stasis.

### Chemicals and perfusates

Perfusates were mixed from stock solutions and solid components on a daily basis and were pH adjusted and sterile filtered. For the composition of perfusates, see Supplementary Table S4. All chemicals were obtained from Sigma-Aldrich (Munich, Germany) if not otherwise stated. Forskolin (CAS: 66575-29-9) was obtained from Cayman Chemical Company (Ann Arbor, United States) and Y-27632 (CAS: 146986-50-7) was obtained from Tocris Bioscience (Bristol, United Kingdom). The CAS registry numbers for the remaining chemicals used were: 2-APB (CAS: 524-95-8); IBMX (CAS: 28822-58-4); ML-7 (CAS: 110448-33-4) and Calphostin C (CAS: 121263-19-2).

### Histological examination and parameters of the model stability

Periodic acid-Schiff (PAS)-stained sections were examined in a blinded fashion. The wet-to-dry weight ratio, the histological stability score and lactate-to-pyruvate ratio at the end of the experiments were assessed and calculated as described before^13^. These data are presented in the Figs 7 and 8, as well as in the Supplementary Fig. S5 and document stable conditions in all experiments. In all the groups the wet-to-dry-weight ratio, the histological stability score and the lactate-to-pyruvate ratio were comparable and in a physiological range. Examples of histological slices for each group are shown in the Supplementary Fig. S1.

### Statistics

Animal numbers were based on a statistical power analysis (1-β) using one-sided t-tests with heterogeneous variances (Proc POWER from SAS) with α = 0.05 and β = 0.2 (a power of 80%) to see 50% improvements compared to PAF for the following parameters: Part, shifts in volume, FITC-dextran and weight gain. The necessary data for the effects of PAF were taken from ref.^13^. These power calculations were confirmed post-hoc (Proc GLMPOWER, data not shown).

All data are given as means ± SD and were plotted by use of Prism software (Graph-Pad Prism version 5.01 for Windows, GraphPad Software, San Diego California, United States). For the statistical analysis of most data the GLIMMIX procedure in SAS 9.4 software (SAS institute GmbH, Heidelberg, Germany) was used. A normal distribution was assumed and confirmed by residual plots for all parameters, except for the histological score (ranging from 0 to 1) for which the beta-distribution was applied. In case of heterogenous variances, we used the Kenward-Roger degrees of freedom approximation (ddfm = KR2). Most data were analysed by one-way ANOVA. In order to examine possible interactions of the various treatments with the fluid or FITC-dextran shifts into either lymph or lumen, these data were analysed by two-way ANOVA with treatment and compartments as the two factors. The α-error was corrected for multiple comparisons by the one-sided step-down Dunnett test, always looking for improvements in comparison to the PAF group.

In order to analyse the entire time courses of the perfusion pressure data within and between groups, we employed non-linear mixed modelling (Proc NLMIXED) using a bi-exponential model with the following terms: k1 reflecting the rapid initial increase, k2 reflecting the slower decay and M reflecting the maximum response:

$$Part=M\frac{{k}_{1}}{{k}_{2}-{k}_{1}}{e}^{-{k}_{1}t}-{e}^{-{k}_{2}t}$$

This approach yields three interpretable parameters that can be statistically compared between the various experimental groups. This procedure is described by Kristensen and Hansen^78^. Multiple comparisons of the obtained p values were corrected using the step-down Bonferroni procedure. P < 0.05 was always considered significant.

## Electronic supplementary material

Supplementary Figure S1: Microscopic images from periodic acid-schiff (PAS)-stained sections.

Supplementary Table S2: Characteristics of the experimental groups.

Supplementary Figure S3: Experimental protocol.

Supplementary Table S4: Composition of perfusates.

Supplementary Figure S5: Lactate-to-pyruvate ratio at the end of the experiments.
