# Supplementary material for: Signalling mechanisms in PAF-induced intestinal failure
Source: Sci Rep. 2017 Oct 17;7:13382. doi: 10.1038/s41598-017-13850-x (PMC5645457; doi:10.1038/s41598-017-13850-x)
Supplement: Supplementary file 1 — Supplementary Figure S1: Microscopic images from periodic acid-schiff (PAS)-stained sections. [file 41598_2017_13850_MOESM1_ESM.doc]

**Supplementary Information**

**Signalling mechanisms in PAF-induced intestinal failure**

Ingmar Lautenschläger, Yuk Lung Wong, Jürgen Sarau, Torsten Goldmann, Karina Zitta, Martin Albrecht, Inéz Frerichs, Norbert Weiler and Stefan Uhlig


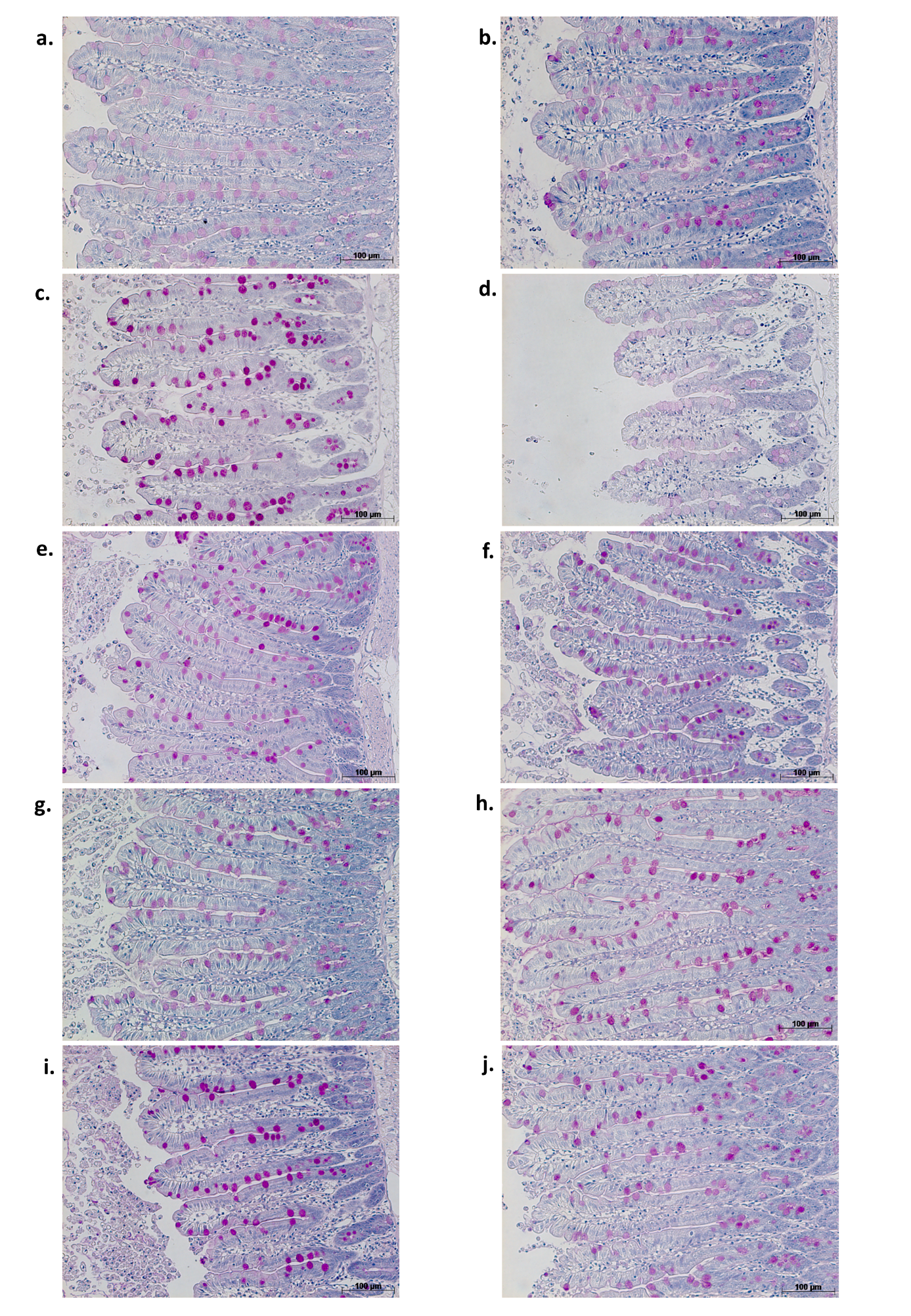
**Supplementary Figure S1: Microscopic images from periodic acid-schiff (PAS)-stained sections.**

Examples of histological longitudinal intestinal slices for each group. All sections were taken from the mesenteric side of the intestinal wall. **a.** PAF group. **b.** Control group. **c.** low Ca+PAF group. **d.** low Ca control group. **e.** 2-APB+PAF group. **f.** Forsk/IBMX+PAF group. **g.** ML-7+PAF group. **h.** Y27632+PAF group. **i.** CalphC+PAF group. **j.** CalphC/Y27632+PAF group.
