# Supplementary material for: Signalling mechanisms in PAF-induced intestinal failure
Source: Sci Rep. 2017 Oct 17;7:13382. doi: 10.1038/s41598-017-13850-x (PMC5645457; doi:10.1038/s41598-017-13850-x)
Supplement: Supplementary file 2 — Supplementary Table S2: Characteristics of the experimental groups. [file 41598_2017_13850_MOESM2_ESM.doc]

**Supplementary Information**

**Signalling mechanisms in PAF-induced intestinal failure**

Ingmar Lautenschläger, Yuk Lung Wong, Jürgen Sarau, Torsten Goldmann, Karina Zitta, Martin Albrecht, Inéz Frerichs, Norbert Weiler and Stefan Uhlig

**Supplementary Table S2: Characteristics of the experimental groups.**

| **Group (n)** | **Agent(s)** | **Amount or concentration** | **Administration** | **Principle** |
| --- | --- | --- | --- | --- |
| low Ca+PAF (3) | - | 0.25 mM | cont. 0-140´ | EC calcium |
| 2-APB+PAF (5) | 2-APB | 50-150 µM | cont. 40-75´ | IP3 calcium |
| Forsk/IBMX+PAF (4) | Forskolin/IBMX | 0.5 µM/100 µM | cont. 40-75´ | cAMP |
| PAF (5) | PAF | 0.5 nmol | bolus | PAF-R |
| control (5) | - | - | - | Control |
| low Ca control (4) | - | 0.25 mM | cont. 0-140´ | EC calcium |
| ML-7+PAF (5) | ML-7 | 35 µM | cont. 40-75´ | MLCK |
| Y27+PAF (3) | Y27632 | 10 µM | cont. 40-75´ | Rho-kinase |
| CalphC+PAF (3) | CalphostinC | 0.5 µM | cont. 40-75´ | PKC |
| CalphC/Y27+PAF (3) | CalphostinC/Y27 | 0.5 µM/10 µM | cont. 40-75´ | PKC/Rho-kinase |

Replicates (n); receptor (R); extra-cellular (EC); 2-Aminoethoxydiphenyl borate (2-APB); inositol-tris-phosphate (IP_3_); 3-isobutyl-1-methylxanthin (IBMX); adenylatcyclase (AC); phosphodiesterase (PDE); myosin light chain kinase (MLCK); protein kinase C (PKC); Y27632 (Y27); continuously (cont.); minute (´); in all experiments (except “control” and “low Ca control”) PAF was given as a bolus of 0.5 nmol at 60´.
