# Supplementary material for: Signalling mechanisms in PAF-induced intestinal failure
Source: Sci Rep. 2017 Oct 17;7:13382. doi: 10.1038/s41598-017-13850-x (PMC5645457; doi:10.1038/s41598-017-13850-x)
Supplement: Supplementary file 3 — Supplementary Figure S3: Experimental protocol. [file 41598_2017_13850_MOESM3_ESM.doc]

**Supplementary Information**

**Signalling mechanisms in PAF-induced intestinal failure**

Ingmar Lautenschläger, Yuk Lung Wong, Jürgen Sarau, Torsten Goldmann, Karina Zitta, Martin Albrecht, Inéz Frerichs, Norbert Weiler and Stefan Uhlig

**Supplementary Figure S3: Experimental protocol.** Bold vertical dashes represent samples for measurement of intestine´s physiology; regular vertical dashes represent samples for additional measurements of FITC dextran transfer; in the groups with low calcium concentration treatment was given during the whole perfusion time; continuously (cont.); minute (´)
