# Supplementary material for: Signalling mechanisms in PAF-induced intestinal failure
Source: Sci Rep. 2017 Oct 17;7:13382. doi: 10.1038/s41598-017-13850-x (PMC5645457; doi:10.1038/s41598-017-13850-x)
Supplement: Supplementary file 4 — Supplementary Table S4: Composition of perfusates. [file 41598_2017_13850_MOESM4_ESM.doc]

**Supplementary Information**

**Signalling mechanisms in PAF-induced intestinal failure**

Ingmar Lautenschläger, Yuk Lung Wong, Jürgen Sarau, Torsten Goldmann, Karina Zitta, Martin Albrecht, Inéz Frerichs, Norbert Weiler and Stefan Uhlig

Supplementary Table S4: Composition of perfusates.

|  | **Vascular perfusate** | **Luminal perfusate** |
| --- | --- | --- |
| Glucose, *mM* | 7.40 | 5.55 |
| Glutamine, *mM* | 0.80 | 0.80 |
| Lactose, *mM* | - | 30.00 |
| NaCl, *mM* | 95.00 | 114.00 |
| KCl, *mM* | 4.70 | 5.00 |
| KH_2_PO_4_, *mM* | 1.20 | - |
| MgSO_4_·7H_2_O, *mM* | 1.20 | - |
| CaCl_2_, *mM* | 2.50 | - |
| Lactobionic acid, *mM* | 2.00 | - |
| Mannitol, *mM* | 30.00 | 10.00 |
| HEPES,  *mM* | 12.60 | - |
| NaHCO_3_, *mM* | 25.00 | 26.00 |
| BSA, *mM* | 450.00 | - |
| pH (adjustment with 2M HCl/NaOH) | 7.52-7.58 | 7.50-7.58 |
| pH after equilibration with carbogen | 7.36±0.02 | - |
| Osmolarity, *mosmol/L* | 310-330 | 310-330 |
| L-Norepinephrine*, *mM* | 5.91·10^-4^ | - |

* Sanofi-Aventis Deutschland GmbH, Frankfurt/Main, Germany.
