# Supplementary material for: Signalling mechanisms in PAF-induced intestinal failure
Source: Sci Rep. 2017 Oct 17;7:13382. doi: 10.1038/s41598-017-13850-x (PMC5645457; doi:10.1038/s41598-017-13850-x)
Supplement: Supplementary file 5 — Supplementary Figure S5: Lactate-to-pyruvate ratio at the end of the experiments. [file 41598_2017_13850_MOESM5_ESM.doc]

**Supplementary Information**

**Signalling mechanisms in PAF-induced intestinal failure**

Ingmar Lautenschläger, Yuk Lung Wong, Jürgen Sarau, Torsten Goldmann, Karina Zitta, Martin Albrecht, Inéz Frerichs, Norbert Weiler and Stefan Uhlig


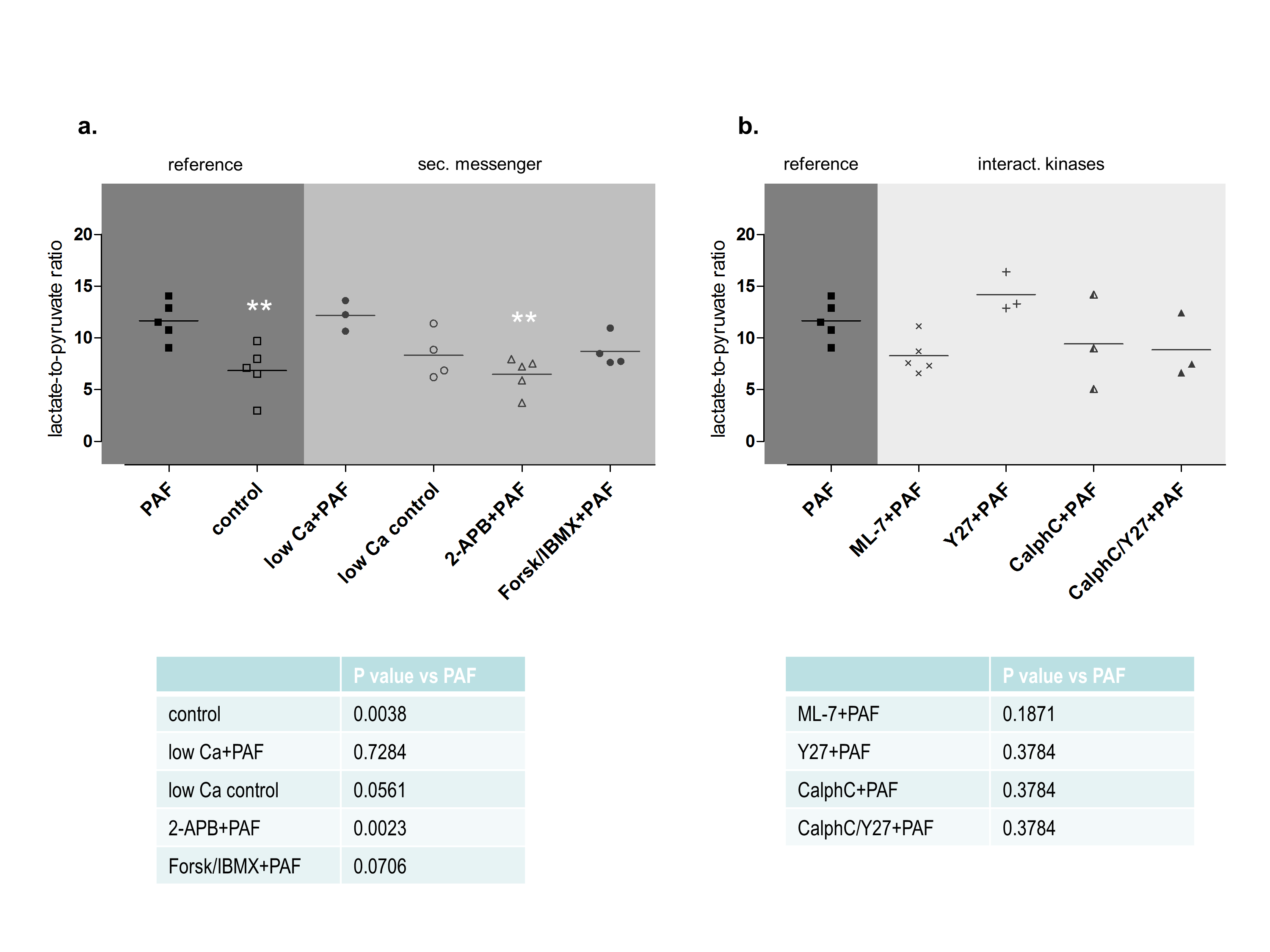
**Supplementary Figure S5: Lactate-to-pyruvate ratio at the end of the experiments. a.** Groups with pretreatment targeting second messengers calcium and cAMP. **b.** Groups with pretreatment targeting MLC interacting kinases. ** p < 0.01 versus PAF. Data are shown as dots plots with median (line).
